# Pathophysiological roles of myristoylated alanine-rich C-kinase substrate (MARCKS) in hematological malignancies

**DOI:** 10.1186/s40364-021-00286-9

**Published:** 2021-05-06

**Authors:** Deepak Narayanan Iyer, Omar Faruq, Lun Zhang, Nasrin Rastgoo, Aijun Liu, Hong Chang

**Affiliations:** 1 Laboratory medicine program, Toronto General Hospital, University Health Network, University of Toronto, Toronto, Canada; 2 Department of Hematology, Beijing Chaoyang Hospital, Capital University, Beijing, China

**Keywords:** MARCKS, Hematological cancers, Drug resistance, Targeted therapy

## Abstract

The myristoylated alanine-rich C-kinase substrate (MARCKS) protein has been at the crossroads of multiple signaling pathways that govern several critical operations in normal and malignant cellular physiology. Functioning as a target of protein kinase C, MARCKS shuttles between the phosphorylated cytosolic form and the unphosphorylated plasma membrane-bound states whilst regulating several molecular partners including, but not limited to calmodulin, actin, phosphatidylinositol-4,5-bisphosphate, and phosphoinositide-3-kinase. As a result of these interactions, MARCKS directly or indirectly modulates a host of cellular functions, primarily including cytoskeletal reorganization, membrane trafficking, cell secretion, inflammatory response, cell migration, and mitosis. Recent evidence indicates that dysregulated expression of MARCKS is associated with the development and progression of hematological cancers. While it is understood that MARCKS impacts the overall carcinogenesis as well as plays a part in determining the disease outcome in blood cancers, we are still at an early stage of interpreting the pathophysiological roles of MARCKS in neoplastic disease. The situation is further complicated by contradictory reports regarding the role of phosphorylated versus an unphosphorylated form of MARCKS as an oncogene versus tumor suppressor in blood cancers. In this review, we will investigate the current body of knowledge and evolving concepts of the physical properties, molecular network, functional attributes, and the likely pathogenic roles of MARCKS in hematological malignancies. Key emphasis will also be laid upon understanding the novel mechanisms by which MARCKS determines the overall disease prognosis by playing a vital role in the induction of therapeutic resistance. Additionally, we will highlight the importance of MARCKS as a valuable therapeutic target in blood cancers and will discuss the potential of existing strategies available to tackle MARCKS-driven blood cancers.

## Background

Since its discovery as a primary substrate of protein kinase C (PKC) [1], the membrane-associated myristoylated alanine-rich C-kinase substrate (MARCKS) (OMIM#: 177061) has been implicated as a watchdog of several cellular processes. From micromanaging actin cytoskeleton dynamics and controlling the molecular role of membrane phosphoinositides, to serving as a critical regulatory node in several signaling networks, MARCKS has been identified to play diverse roles within multiple cell systems, tissues, and organs. Specifically, within the hematological system, MARCKS is associated with the regulation of body fluid homeostasis, coagulation, cell motility, vesicular trafficking, cell proliferation, secretion, and mediation of inflammatory response. Not surprisingly, several recent studies have identified a deregulated expression of MARCKS to be associated with abnormal cellular physiology and severe pathological outcomes [2–10]. Moreover, with MARCKS being commonly associated with numerous oncogenic signaling pathways, aberrant expression of MARCKS, and more recurrently its phosphorylated form, has been frequently reported in several solid cancers [4, 11–13]. Within blood cancers, although there are several studies that have identified a potent role of deregulated MARCKS with the overall process of carcinogenesis [14–18], yet our overall understanding of the regulatory roles of the gene and the associated protein remains at a nascent stage. Whereas some hematological malignancies have stressed the importance of the phosphorylated form of MARCKS in cancer development [14, 18, 19], others specify the involvement of the unphosphorylated protein in governing disease outcomes [15]. Additionally, while MARCKS exists as a valuable candidate for anti-cancer therapies, currently our grasp on the strategies available to target the gene or protein is still evolving. Notwithstanding the lag, blood cancer exists as a particularly valuable system that is experimentally tractable and hence can be used to investigate the pathobiology of MARCKS, also giving a prospect to reverse its adverse impact in cancer.

In this review, we focus on the functional importance of MARCKS in normal and cancer physiology, with an explicit emphasis on hematological cancers. We will summarize the current knowledge of the signaling networks regulated by MARCKS that may directly or indirectly impact its role on the development, progression, and overall outcome of the disease. Finally, we will discuss the potential therapeutic strategies for addressing the pathological consequences of aberrant MARCKS signaling in hematological cancers.

## Structural consequence on protein localization

### Protein structure

**Fig. 1:**
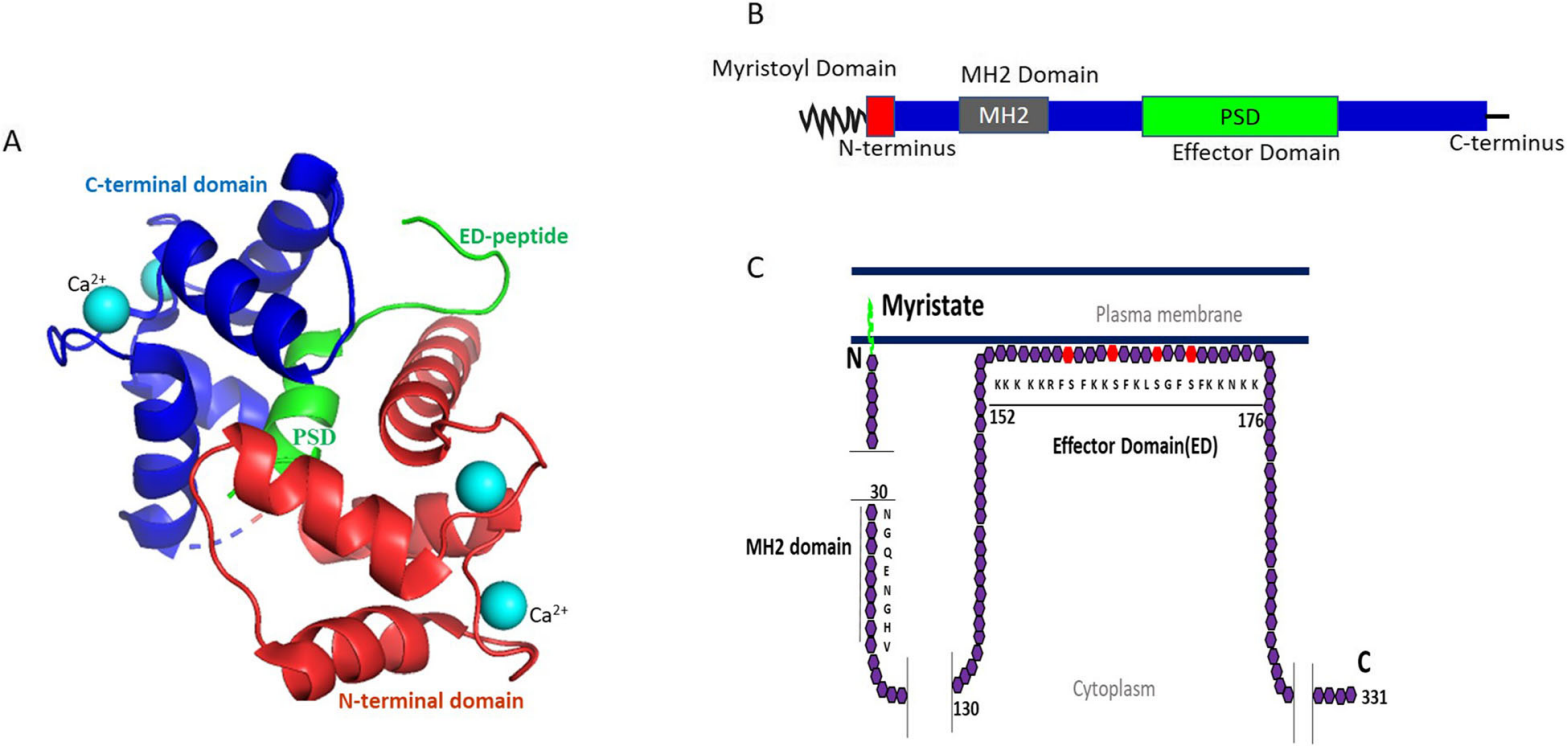
MARCKS protein structure. **(a)** The image was created from the protein data bank (PDB) entries 1IWQ [22] and adapted using PyMOL Version 2.2 [23]. **(b)** Linear representation of the MARCKS protein containing the Myristoyl Domain, N-terminal Domain, MH2 domain, a phosphorylation site domain (PSD; also known as the effector domain (ED)), and C-terminal Domain (**c**) Schematic representation of the MARCKS protein bound to the plasma membrane by the myristoylated N-terminal domain. Other structural elements include an MH2 domain and the ED. The ED (amino acids: 152–176) can be phosphorylated by PKC at three or four serine residues (marked in red) or can be bound to CaM and actin. The ED also electrostatically interacts with the plasma membrane and provides additional support to the N-terminal myristate moiety-mediated binding of MARCKS to the phospholipid bilayer of the membrane

MARCKS is a ubiquitous, rod-shaped, 32 kDa protein that is a single gene product and exists in the category of ‘natively unfolded’ proteins with little apparent secondary structure [20]. The protein is highly acidic owing to an unusually rich concentration of alanine, proline, glycine, and glutamic acid residues [21]. Consequently, MARCKS exhibits thermal stability and anomalously slow-migration on an SDS-PAGE gel at an apparent molecular weight of 80–87 kDa. Owing to this behavior, Wu et al. had first described MARCKS as the “87k protein” when he discovered it in 1982 as a substrate for phosphorylation by calcium/phospholipid-dependent PKC [1]. Structurally, MARCKS is composed of three highly conserved regions (Fig. 1). The *N-*terminal domain that has a consensus sequence (H_2_N-GXXXS) for a reversible, co-translational, covalent attachment of a 14-carbon saturated myristoyl chain to the amino-terminal glycine residue, a process catalyzed by myristoyl-CoA:protein N-myristoyltransferase (NMT) [24]. The second structural component is the MARCKS Homology 2 (MH2) domain that bears similarity with the cytoplasmic tail of the cation-independent mannose-6-phosphate receptor (CI-MPR), surrounds the site of splicing of the sole intron in the genes for MARCKS family of proteins, yet the function of this domain remains unknown [20, 25]. Finally, central to the function of MARCKS protein is the phosphorylation site domain (PSD), since it is composed of all the target serine residues for a PKC-mediated phosphorylation event and is consequently referred to as the effector domain (ED). Several studies have described that the PSD can also serve as a substrate for phosphorylation by other protein kinases such as Rho-associated kinase (ROCK) [26, 27]. It is worth mentioning here that the only other member of the MARCKS family of proteins is the 20 kDa MARCKS-related protein (MRP, also known as MARCKS-like protein (MLP), MARCKS-like protein 1 (MARCKSL1), Brain Protein F52, or MacMARCKS) that shares a strong structural (96% sequence homology) and functional homology with MARCKS [28, 29].

### Membrane versus cytosolic localization

The electrostatic interaction between the highly basic PSD and the heavily acidic residues within the rest of the protein causes the N-terminal myristoyl moiety to hydrophobically embed itself within the lipid bilayers [30]; the collaborative effect (electrostatic switch & myristoyl embedding) ensuing the stabilized membrane binding of MARCKS [31, 32]. Phosphorylation of PSD by PKC causes the attachment of serine residues with negatively charged phosphate groups which results in the neutralization of the domain’s positive charges and obliterates the electrostatic switch. Because myristoylation on its own is a weak force to tether the protein to the membrane, phosphorylated MARCKS is detached from the plasma membrane and is translocated to the cytoplasm [33, 34]. Additionally, there is some evidence to indicate that phosphorylated MARCKS may also translocate to the nucleus, although its role within the nucleus requires further exploration [35, 36]. On the contrary, dephosphorylation of the PSD by protein phosphatase 1, protein phosphatase 2A or calcineurin results in the reassociation of MARCKS with the membrane [37–39]. Put together, the reversible translocation of MARCKS from the plasma membrane to the cytoplasm, and the resulting downstream molecular impact of this action, is dependent primarily on the phosphorylation-dephosphorylation cycles of the MARCKS protein. An alternative molecular basis for the reversible translocation of MARCKS can be attributed to the association of ED with Calmodulin (CaM); subject to intracellular calcium mobilization [40]. In the presence of a high intracellular concentration of calcium, the MARCKS protein can also be activated and subsequently be disassociated from the plasma membrane, by reversibly binding to CaM. Once the intracellular calcium is returned to normal levels, MARCKS is released from CaM and shuttles back to the plasma membrane.

Importantly, the two activation pathways – PKC based (phosphorylation-dependent) or CaM based (calcium-dependent) – are incongruous mechanisms that attempt to overpower each other, albeit with a mutual aim of activating MARCKS [40]. CaM bound to MARCKS has been reported to cause a steric hindrance for any phosphorylation event by PKC [20]. In contrast, phosphorylation of the ED was reported to cause a nearly 200-fold decrease in the affinity of the domain for CaM, in addition to disrupting any pre-existent MARCKS-CaM complexes [41]. Consequently, Blackshear suggested that on one hand MARCKS could channel the release of CaM and serve as an intracellular reservoir for the protein in cells lacking any PKC activity [25]. Although, as a key substrate of PKC, MARCKS also serves as a vital intracellular CaM donor [25]. Interestingly, an investigation by Yamamoto et al. demonstrated that in contrast to the effect of PKC, phosphorylation of MARCKS by proline-directed kinases such as cyclin-dependent kinase 1 (CDK1(cdc2a)), and tau protein kinase II (TPKII) encouraged CaM binding to MARCKS [42]. Moreover, while PKC was found to specifically target the seryl residues, both serine and threonine residues were phosphorylated in the presence of CDK1 and TPKII [42]. The precise molecular mechanisms remain complex and require further investigations to understand the critical processes associated with MARCKS activation.

## Functional impact of molecular interactions

### Molecular partner: actin

**Fig. 2:**
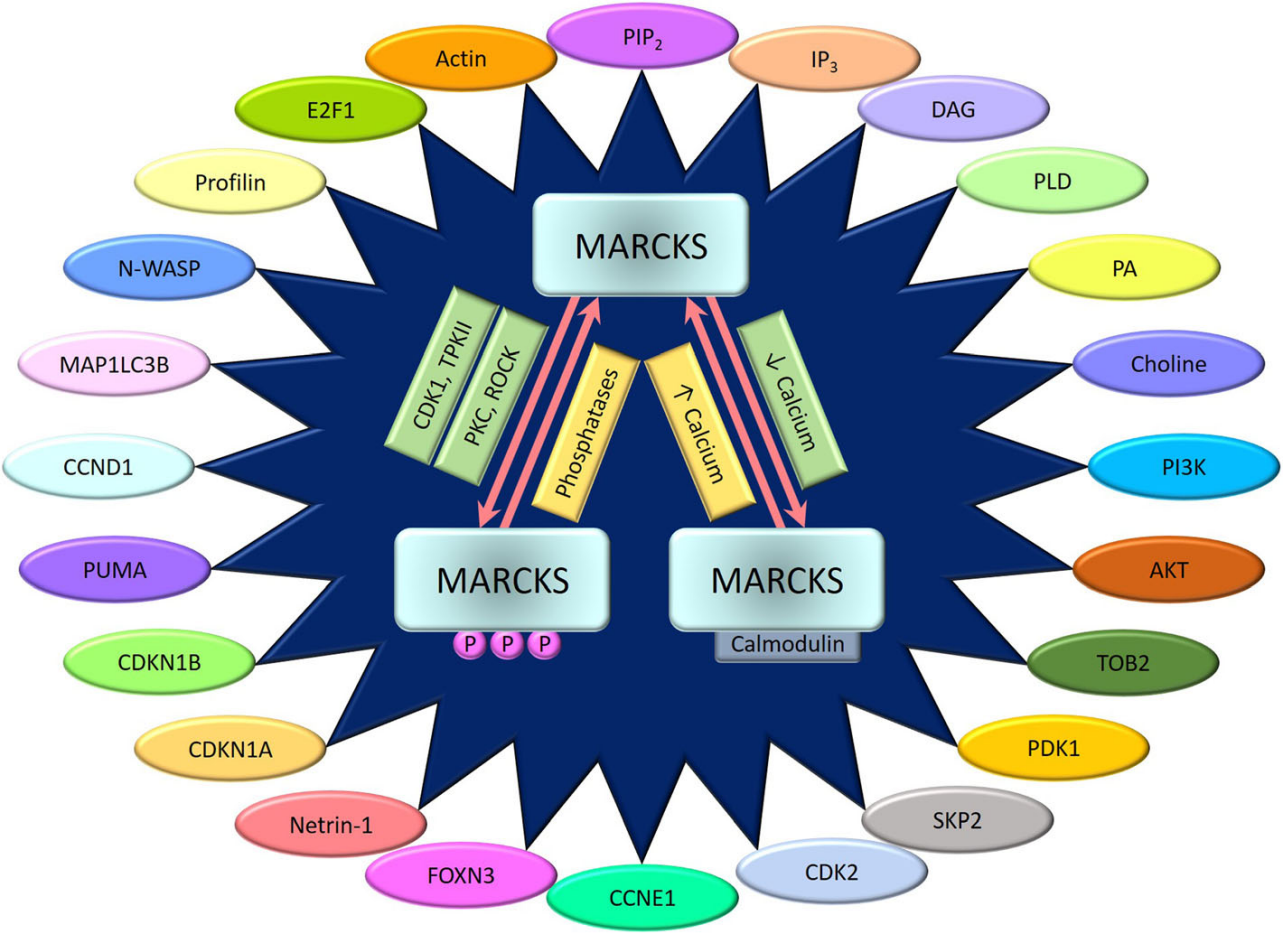
Molecular partners of MARCKS. While MARCKS shuttles between the phosphorylation-dependent or calcium-dependent activation pathways, it directly or indirectly modulates the activity of several key members within multiple signaling networks. Shown are the major molecular partners that contribute to the functional relevance of MARCKS

Amid the activation-inactivation cycles of MARCKS which consequently determine its membrane versus cytosolic localization, the protein has been shown to engage in critical molecular interactions (Fig. 2). Inactivated MARCKS protein localized to the plasma membrane directly binds to and cross-links filamentous actin (F-actin) through two separate actin-binding sites, while this activity is inhibited by the phosphorylation of MARCKS or its binding to CaM [43–45]. Indeed, phosphorylation of MARCKS was shown to induce structural changes within the active site of the protein that impede efficient actin filament cross-linking [46]. Interaction of MARCKS with actin was more specifically observed during cell adhesion, membrane ruffling, and cell spreading, signifying the protein’s role in regulating the cytoskeleton in critical biological processes such as wound healing, morphogenesis, embryogenesis, and metastasis [47]. Like MARCKS, the ED of MRP has been shown to efficiently bundle actin filaments besides inducing rapid polymerization of monomeric actin into filaments [48]. However, a later study published by the same research group using the intact MRP demonstrated that while the protein can bind F-actin with micromolar affinity, it does so without significantly impacting actin polymerization or any cross-linking activity [49]. Further data is essential to understand the interactions of MARCKS proteins with actin and its associated physiological outcomes.

### Molecular partner: Phosphatidylinositol-4,5-bisphosphate

A significant proportion of the cellular functions of MARCKS are executed through its interactions with the phosphatidylinositol-4,5-bisphosphate (PIP_2_). PIP_2_ is a low abundance polyphosphoinositide that represents less than 1% of membrane phospholipids, yet plays a crucial role in several cellular processes including membrane trafficking, membrane attachment to cytoskeleton, phagocytosis, stabilization or activation of membrane ion channels and transporters [50], and intracellular signaling [51, 52]. Moreover, PIP_2_ has also been identified to regulate actin-binding proteins such as Neural-Wiskott-Aldrich Syndrome Protein (N-WASP) [53] and Profilin [54, 55]; thereby representing an indirect MARCKS-free regulation of actin assembly. Hydrolysis of PIP_2_ by phospholipase C (PLC) facilitates the production of secondary messenger molecules inositol-1,4,5-trisphosphate (IP_3_) and diacylglycerol (DAG) which ultimately lead to the release of intracellular calcium stores and activation of PKC [56]. Several studies have shown that the MARCKS protein, specifically through its PSD, sequesters PIP_2_ to the cell membrane by non-specific electrostatic interactions [57–60]. The formation of PIP_2_-MARCKS (151–175) complexes within specific membrane micro-domains or “lipid rafts” encumbers the hydrolysis of PIP_2_ by inducing the inhibition of PLC [61]. This inhibition can however be reversed by PKC or CaM, either of which can bind the MARCKS-PSD peptide, causing the release of PIP_2_ [62].

The control of PIP_2_ allows MARCKS to regulate the activation of secondary messengers IP_3_ and DAG along with several other downstream signaling components. Phospholipase D (PLD) is one such molecule whose activity, membrane localization, and receptor activation are stringently regulated by PIP_2_ [63–65], and indirectly by MARCKS. The primary substrate for PLD is phosphatidylcholine (PC) which is hydrolyzed to generate phosphatidic acid (PA) and choline which serve as secondary messengers themselves [66–69]. While the cell-signaling molecule PA can be further metabolized to lysophosphatidic acid (LPA) and DAG [70], choline can be metabolized to phosphocholine which functions as a regulator of cell proliferation [71]. These secondary messengers allow PLD, and indirectly MARCKS, to regulate multiple cellular processes including cell migration, membrane trafficking, mitosis, vesicular trafficking, receptor endocytosis, exocytosis, and cytoskeletal reorganization [72, 73]. A recent report by Ziemba et al. demonstrates that MARCKS potentially regulates the activation and function of phosphoinositide-3-kinase (PI3K) by controlling the bioavailability of free PIP_2_ [74]. Using single-molecule fluorescence, the authors investigated the Ca(2+)−PKC-MARCKS-PIP2-PI3K-PIP3 amplification module and observed that in the off state of the module, MARCKS sequesters PIP_2_ to the cell membrane and prevents its availability as a docking target and a substrate lipid for PI3K. Under the influence of Ca^2+^-PKC phosphorylation, PIP_2_ is released from membrane sequestration and is available for phosphorylation by PI3K that subsequently promotes the membrane recruitment and activation of cytosolic proteins phosphoinositide-dependent kinase-1 (PDK1) and Akt, thereby initiating the PI3K/Akt signaling network [74–76]. Consequently, through the PIP_2_-dependent PI3K/Akt pathway, MARCKS plays a major role in regulating a myriad of cellular processes including cell proliferation, survival, growth, and death.

### Other molecular partners

Apart from actin and PIP_2_, MARCKS affects several other signaling pathways through multiple mechanisms. Using the MDA-MB-231 human breast cancer cell line model, Cho et al. demonstrated that phosphorylation of the MARCKS ED induced the activation of the proto-oncogene receptor protein tyrosine kinase (PTK) ErbB-2 (also known as HER2) by binding to antiproliferative protein Transducer of ERBB2, 2 (TOB2) that regulates cell cycle by inhibiting progression from the G0/G1 to S phases [77]. Interestingly, ErbB-2 has been found to be frequently overexpressed and associated with a poor disease outcome in several human malignancies [78]. Likewise, another cancer-associated pathway [79, 80] modulated by MARCKS is the Netrin-1 signaling through the Deleted in Colorectal Cancer (DCC) receptor [81]. Brudvig et al. further establishe that in the absence of MARCKS, the subcellular distributions of the tyrosine kinases PTK_2_ and SRC which function as regulators of NTN1-DCC signaling are disrupted [81].

### Role in the hematological system

Among several other functions, MARCKS protein has been identified as a vital controller of secretion in various cell types. Phosphorylated MARCKS has been reported to induce thrombin-induced serotonin release in platelets [82], synaptic vesicle trafficking and neurotransmitter release by neuronal cells [83, 84], adrenocorticotropin secretion from the ovine anterior pituitary [85], and mucin secretion in human airway epithelial cells [86]. Moreover, phosphorylated MARCKS has a recognized role in the migration of inflammatory leukocytes such as neutrophils and macrophages, besides secretion of inflammatory cytokines [87–90]. Furthermore, PKC-phosphorylated MARCKS in macrophages was observed to regulate phagocytosis through actin-dependent formation, maturation, and translocation of phagosomes [91].

Indeed, within the hematological system, several such regulatory roles of MARCKS have been observed. By modulating the availability of free PIP_2_, MARCKS or MRP adjusts the epithelial sodium channel open probability that has a remarkable role in the regulation of total body fluid homeostasis and blood pressure control [92, 93]. Furthermore, MARCKS ED-based peptides have been reported to bind to physiologically exposed phosphatidylserine on activated platelets and play a critical role in regulating the coagulation cascade [94]. Another report indicates that MARCKS moderates PIP_2_ signaling to shift between microtubule-driven processes like proplatelet extension versus actin-related protein 2/3 and actin polymerization-controlled processes such as proplatelet branching [95]. Supporting the anticoagulatory role of MARCKS, Chu et al. used model human leukemia THP-1 monocytes to demonstrate that MARCKS has a possible function in blocking monocytic tissue factor initiated hypercoagulation [96]. MARCKS has also been well-characterized as a regulator of motility in macrophages, neutrophils, and other cell types in both, its unphosphorylated [97, 98] and cytosolic forms [11, 99, 100]. While this is an indicator of the existence of context-dependent roles of MARCKS, it is also suggestive of a dynamic cellular shuffling between the phosphorylated and unphosphorylated versions of the protein [101]. For example, during dramatic cellular morphological changes that occur throughout cell migration and tissue morphogenesis, Disatnik and group demonstrate the existence of a bi-directional translocation of MARCKS, with the cytosolic MARCKS promoting the early phases of cell adhesion, while the dephosphorylated, membrane-associated MARCKS being responsible for later stages of cell spreading [45].

Taken together, while there is a significant lack in our understanding of the structural and functional significance of MARCKS, the information that we have until now makes MARCKS a potent guardian of the cell regulating innumerable processes and molecular pathways in normal physiology.

## MARCKS impacts the development and progression of blood cancers

Whereas the functional influence of MARCKS on cellular systems extends beyond developmental and maintenance workflow, numerous studies have now indicated that anomalous expression of MARCKS is often associated with several cancers. Extensive data suggest that dysregulated MARCKS expression drives the development and progression of several solid tumors including melanoma [11, 102, 103], glioma [12, 104, 105], renal cell carcinoma [106], lung cancer [107–109], colorectal cancer [110, 111], liver cancer [13, 112], and breast cancer [113–115]. Similarly, aberrant MARCKS has been observed to contribute to increased cell proliferation, reduced cell death, higher rates of cell migration, invasion, and motility, and malignant transformation in several hematological malignancies (Table 1). One of the earliest studies identifying an association between the deregulated expression of MARCKS with the development of blood cancers was by Nagata et al. [118]. Using MEG-01, a model human megakaryoblastic leukemia cell line, the authors reported that different PKC isozymes impacted the initiation and maintenance of differentiation of the leukemia cells by regulating the phosphorylation and membrane localization of MARCKS. In another early study, the authors incubated B-chronic lymphocytic leukemia (B-CLL) cells with phorbol esters that resulted in the phosphorylation of MARCKS, MRP, and a third novel MARCKS-like protein, subsequently characterized as lymphocyte-specific protein 1 [121, 122]. Bearing a remarkable physiological and pathological similarity to MARCKS, the lymphocyte-specific protein 1 harbors phosphorylation sites for serine/threonine kinases, in addition to actin-binding sites for interacting with F-actin, and regulates neutrophil chemotaxis during inflammation [133], and is also frequently overexpressed in a wide range of lymphomas and leukemias [122, 134, 135].

**Table 1:** Role of MARCKS in hematological malignancies

Blood Cancer Subtype	Role of MARCKS in	Critical MARCKS-related outcomes	Reference
Acute Myeloid Leukemia (AML)	Disease signature Disease progression	MARCKS is associated with receptor tyrosine kinase TrkA and KIT expression and is a marker of poor outcome in AML.	[116]
Chronic Myelogenous Leukemia (CML)	Disease development Disease signature	Evidence of alternative splicing in MARCKS was identified in leukemic stem cells in CML.	[117]
Chronic Myelogenous Leukemia (CML)	Disease development	MARCKS plays an important role in the differentiation process of human megakaryoblastic leukaemia cell line MEG-01 through its interaction with PKC.	[118]
Chronic Myelogenous Leukemia (CML)	Drug Target	Treatment of HUVECs with exosomes derived from CML cells treated with curcumin alone or enriched with miR-21 reduced MARCKS expression significantly.	[119]
Myeloid malignancies	Disease development Disease progression	NADPH oxidase signaling may be mediated through MARCKS phosphorylation of ED in myeloid malignancies.	[120]
Acute Lymphoblastic Leukemia (ALL)	Drug resistance Disease progression	(1) MARCKS is associated with poor prognosis in therapy-refractory leukemia patients, specifically treated with bortezomib. (2) MARCKS is responsible for formation and exocytosis-mediated extrusion of ubiquitin-containing vesicles in bortezomib-resistant leukemic cells, reducing cellular proteasomal load, promoting cell-survival.	[15]
Chronic Lymphocytic Leukemia (CLL)	Disease occurrence	Incubation of B-CLL cells with phorbol esters resulted in the phosphorylation of PKC substrates MARCKS, MRP and a novel protein of apparent 60 kDa molecular weight.	[121]
Chronic Lymphocytic Leukemia (CLL)	Disease occurrence	Incubation of B-CLL cells with phorbol esters resulted in the phosphorylation of PKC substrates MARCKS, MRP and a novel protein of apparent 60 kDa molecular weight, subsequently characterized as lymphocyte-specific protein 1.	[122]
Mantle Cell Lymphoma (MCL) & Chronic Lymphocytic Leukemia (CLL)	Disease signature	(1) MARCKS is differentially expressed, localized and phosphorylated between MCL and CLL. (2) Oncogenic miR-155 inhibits MARCKS expression in CLL. (3) MARCKS has an important role in the MCL pathogenesis and can function as an MCL biomarker.	[19]
Mantle Cell Lymphoma	Disease signature	MARCKS is upregulated in the Blastoid Variant of Mantle Cell Lymphoma.	[123]
Mantle Cell Lymphoma	Disease signature	MARCKS is less expressed in Mantle Cell Lymphoma with low levels of the long cyclin D1 transcript as compared to other MCL with a higher expression of cyclin D1 variant.	[124]
Burkitt’s Lymphoma (BL)	Disease occurrence	MARCKS is one of the previously unknown genes found to be upregulated in Epstein-Barr virus infected B-lymphocytes.	[125]
B-cell lymphoma	Disease occurrence	Type-1 Epstein-Barr virus antigen 2 causes a significant induction of MARCKS in lymphoblastoid cell lines as compared to type-2 Epstein-Barr virus antigen 2.	[126]
Lymphoplasmacytic Lymphoma (Waldenström’s macroglobulinemia)	Disease signature	LEF1, MARCKS, ATXN1 and FMOD form a gene signature that can discriminate clonal B-lymphocytes from Waldenström’s macroglobulinemia and chronic lymphocytic leukemia	[127]
Lymphoplasmacytic Lymphoma (Waldenström’s macroglobulinemia)	Therapeutic target	Protein kinase C inhibitor Enzastaurin inhibits phosphorylation of MARCKS and other signaling molecules downstream of PKC, and subsequently induces anti-tumor activity in vitro and in vivo in Waldenström’s macroglobulinemia.	[16]
Diffuse large B cell lymphoma (DLBCL)	Disease progression Drug resistance	6q21 (near MARCKS and HDAC2 genes) was identified as one of the top loci marked with rs7765004 genetic variant associated with event-free survival and overall survival in patients with DLBCL.	[128]
Diffuse large B cell lymphoma (DLBCL)	Disease progression Drug resistance	Immunohistochemical staining shows a higher expression of MARCKS-like protein in DLBCL patients who remain progression-free for more than 5 years following initial diagnosis.	[129]
B-Cell tumor	Disease occurrence	(1) Unphosphorylated MARCKS suppressed proliferation and survival of B-cell tumor cells and splenic B cells in vitro and in vivo. (2) MARCKS regulates strength of B-cell signaling by modulating cytoskeleton and plasma membrane interactions.	[130]
T-cell Lymphoma	Radiation resistance Disease progression	Frequent mutations were observed in MARCKS in spontaneous and infrared-radiation induced lymphomas in mice models with biallelic germline mutations in DNA mismatch repair gene MLH1.	[17]
Multiple Myeloma	Drug resistance Disease progression	PKC-inhibitor enzastaurin inhibits phorbol ester-induced phosphorylation of MARCKS and other downstream signalling molecules.	[131]
Multiple Myeloma	Drug resistance Disease progression Therapeutic target	(1) MARCKS is overexpressed in drug-resistant myeloma. (2) Knockdown of MARCKS or inhibition of phosphorylation enhanced therapeutic sensitivity.	[14]
Multiple Myeloma	Drug resistance Disease progression	(1) Jagged1 induced activation of Notch-PKC pathway in myeloma cells causes MARCKS to play vital roles in the development of drug-resistant myeloma cells. (2) The PKC-MARCKS pathway is a vital druggable target in refractory multiple myeloma.	[18]
Multiple Myeloma	Drug resistance Disease progression	(1) miR-34a regulates MARCKS expression. (2) Combining traditional chemotherapy with MARCKS antagonists increases effectiveness against drug resistant MM cells	[132]

### Association with predisposing factors

MARCKS has also been observed to possibly contribute to some risk factors that predispose the development of several blood cancers. Frequent frameshift mutations were identified in mononucleotide repeat sequences within *MARCKS* in spontaneous and infrared-radiation associated T-cell lymphomas with biallelic germline mutations in the DNA mismatch repair gene *MLH1* [17]. Germline mutations in the human mismatch repair genes such as *MLH1* and *MSH2* have been reported to increase the likelihood of pediatric T-cell leukemias and lymphomas [136–138]. Inter alia, Epstein-Barr virus (EBV) is a recognized risk factor for several lymphomas. Although more than 90% of adults carry lifelong latent EBV infection in B-lymphocytes, the immunocompetent hosts harbor infected B-cells in a resting stage with no clinical manifestations [139, 140]. In individuals with immune dysfunction resulting from a profound T-cell impairment, EBV may cause infectious mononucleosis, a benign proliferation of B-lymphocytes that can lead to acute EBV-positive B-lymphoproliferative disease [141, 142]. In order to identify activation effects of EBV infection in primary B-lymphocytes, Birkenbach et al. used subjective hybridization to identify genes that are differentially expressed between EBV-positive Burkitt Lymphoma (BL) cells versus the EBV-negative BL cells [125]. Among other novel findings, the authors discovered a 30-fold induction of MARCKS mRNA within the EBV-positive BL cells, suggesting that increased MARCKS expression may be an outcome of EBV-activating and differentiating effect on B-lymphocytes [125]. A subsequent study stated that MARCKS activation is induced significantly in the presence of type-1 EBV nuclear antigen 2 (EBNA2) as compared to type-2 EBNA2 in lymphoblastoid cell lines (LCL) [126]. Interestingly, a type-1 variant of EBNA2 has been reported to function as a transcription factor inducing the expression of viral latent membrane protein (LMP) genes and other genes that regulate cell proliferation and survival in LCLs, at a significantly greater efficacy than the type-2 variant of EBNA2 [143]. Furthermore, EBV proteins LMP1 and LMP2A are well-documented activators of the oncogenic PI3K/Akt signaling pathway that subsequently promotes carcinogenesis [144–146]. Since MARCKS is a known regulator of PI3K signaling, it may have a further role in advancing the EBV-induced effects of increasing cell proliferation, genomic instability, and decreasing cell death in EBV-associated lymphomas.

### MARCKS: an oncogene or a tumor suppressor?

While MARCKS regulates a plethora of processes that impact the oncogenic potential across several cell types, the role of MARCKS as a cancer promoter or a tumor suppressor is still not clearly ascertained. Within solid cancers, MARCKS has commonly been identified with a cancer-promoting role in breast cancer [113–115], lung cancer [107–109], melanoma [11, 103], glioma [104], renal cell carcinoma [106], and liver cancer [13, 112]. Contrarily, several research groups have observed that higher expression of MARCKS inhibits cancer development and progression in colorectal cancer [110, 147], hepatocellular carcinoma [148], melanoma [149], and glioma [12, 105, 150]. Such a dual role of MARCKS has been observed in blood cancers as well. Several reports have identified MARCKS as a positive regulator of the carcinogenesis process in multiple hematological malignancies. In patients with mantle cell lymphoma (MCL), specifically, the Ser159/163 phosphorylated form of MARCKS has been reported to be upregulated [19]. Similarly, Sven and colleagues also report an increase in the expression of MARCKS in the blastoid variant of MCL, a rare, aggressive form of non-Hodgkin’s lymphoma (NHL) [123]. However, conflicting evidence from a few studies has shown MARCKS to be associated with a growth-inhibitory role in cancer. A report by Sanders and colleagues found that the expression of *MARCKS* was lower in MCL disease expressing low levels of full-length cyclin D1 transcript variant [124]. Remarkably, decreased expression of long cyclin D1 4.4 kb transcript is commonly associated with high proliferation rates and poor survival in MCL [151, 152]. Likewise, Vargova et al. identified a nearly four-fold downregulated expression of MARCKS in CLL as compared to the normal control subjects [19]. Mechanistically, the authors observed that a greater expression of the oncogenic miR-155 in CLL caused a sequence-specific suppression of the expression of *MARCKS* thereby controlling its cellular levels. Studies indicate that miR-155 is proinflammatory and is associated with several physiological processes including hematopoiesis, immunity, and cell lineage differentiation [153]. Deregulated expression of miR-155 is consequently linked with an aggressive disease presentation and an overall poor prognosis in diffuse large B-cell lymphoma (DLBCL), acute myeloid leukemia (AML), and CLL [153–156]. Such conflicting reports regarding the behavior of MARCKS have been reported in other blood cancers as well. Using a genome-wide association study, Ghesquieres and his group identified a novel single nucleotide polymorphism rs7765004 near the chromosomal location of *MARCKS* which was found to be associated with poor event-free survival and overall survival in patients with DLBCL treated with immunochemotherapy [128]. Contrastingly, a study by Ednersson and colleagues showed a higher expression of MARCKS protein within DLBCL patients showing a longer disease-free survival period as compared to patients with refractory disease or early relapse [129]. Although the authors did not provide any mechanistic hypothesis, such contradictory observations are possibly an outcome of context-dependent regulation of MARCKS, its phosphorylation status and its membrane versus cytosolic localization. Moreover, further genetic evidence is required to have a stringent classification of MARCKS’s role in promoting or suppressing tumorigenesis. Consequently, a deeper investigation is essential for us to better understand the role of MARCKS in normal and cancer physiology.

## MARCKS affects disease outcomes in blood cancers

In addition to its role in the development and progression of hematological malignancies, MARCKS has been implicated in defining the overall outcome in several blood cancers. A high expression of *MARCKS* has been reported to be associated with an overall poor disease prognosis in AML [116]. Research from our lab investigating the role of MARCKS in hematological malignancies provided the earliest evidence of its involvement in drug-resistant multiple myeloma (MM), chiefly against the proteasomal inhibitor bortezomib [157]. Specifically, our data discovered an increase in the expression of total, and more precisely the phosphorylated form of MARCKS in drug-resistant MM cell lines and MM patients showing disease relapse [14]. Furthermore, by inhibiting the phosphorylation or overall expression of MARCKS, an increase in the sensitivity of MM cell lines to different classes of anti-myeloma drugs including proteasomal inhibitor bortezomib, corticosteroid dexamethasone, anthracycline antibiotic doxorubicin and immunomodulatory drug lenalidomide was observed. Inhibition of MARCKS expression was also found to be associated with an increased expression of proapoptotic genes including Cyclin-dependent kinase inhibitor 1B (CDK1B/p27^kip1^), Cyclin-dependent kinase inhibitor 1A (CDKN1A/p21^Cip1^), Cyclin D1 (CCND1), and p53 upregulated modulator of apoptosis (PUMA), with a concurrent decrease in the levels of antiapoptotic genes S-phase kinase-associated protein 2 (SKP2), Cyclin-dependent kinase 2 (CDK2), G1/S-specific cyclin-E1 (CCNE1) and Forkhead box protein N3 (FOXN3). Mechanistically, the phosphorylated form of MARCKS was observed to associate with the transcription factor E2F1 to directly regulate the SKP2/P27 axis in promoting cell cycle progression and inhibiting apoptosis in drug-resistant MM cells. A subsequent study by Muguruma et al. corroborated our findings and further demonstrated a unique molecular mechanism that involves the bone marrow myeloma niche cells-localized Jagged1-induced activation of the Notch/PKC pathway that subsequently phosphorylates MARCKS and enhances its involvement in the development of drug-resistance in MM [18].

The role of MARCKS in the development of drug resistance was observed in other blood malignancies as well. Franke and colleagues reported an upregulated expression of MARCKS in bortezomib-resistant leukemia cells as well as refractory pediatric leukemia patients treated with bortezomib-based chemotherapy [15]. An increase in the expression of MARCKS was also observed in leukemia cells showing acquired resistance to other second-generation proteasomal inhibitors including, Salinosporamide A (Marizomib) and the immunoproteasome inhibitor PR924. At the molecular level, MARCKS was observed to facilitate exocytosis-mediated extrusion of polyubiquitinated proteins to compensate for the proteolytic stress imposed by bortezomib and other proteasomal inhibitors. By disposing of the abundant ubiquitinated proteins MARCKS was observed to prolong therapeutic resistance by possibly circumventing unfolded protein response which is also a common occurrence in other bortezomib-resistant blood cancers [158–160]. Of note, unlike MM, phosphorylated MARCKS displayed a low basal expression in leukemia cells selected for drug resistance [15]. Additionally, stimulated changes to the phosphorylation status of MARCKS also had no impact on the sensitivity of leukemia cells towards bortezomib treatment. Contrastingly, unphosphorylated MARCKS was not only upregulated in leukemic cells exposed to bortezomib, rather it co-localized with ubiquitin in exocytosed vesicles. Indeed these observations are highly inconsistent with the conclusions drawn by Fong et al in their recent review wherein they suggest that whereas phosphorylated MARCKS promotes cancer cell survival and proliferation by potentiating PIP_2_-dependent PI3K/Akt pathways, unphosphorylated MARCKS, especially at an increased level, behaves as a tumor suppressor [4]. Although such observations may be labeled as being context-dependent, the extremely contradictory nature of the reported roles of unphosphorylated and phosphorylated MARCKS in such cases requires significant follow-up investigation to further our understanding of the roles of MARCKS in cancer development and progression.

## Chemotherapeutic potential of MARCKS in blood cancers

**Fig. 3:**
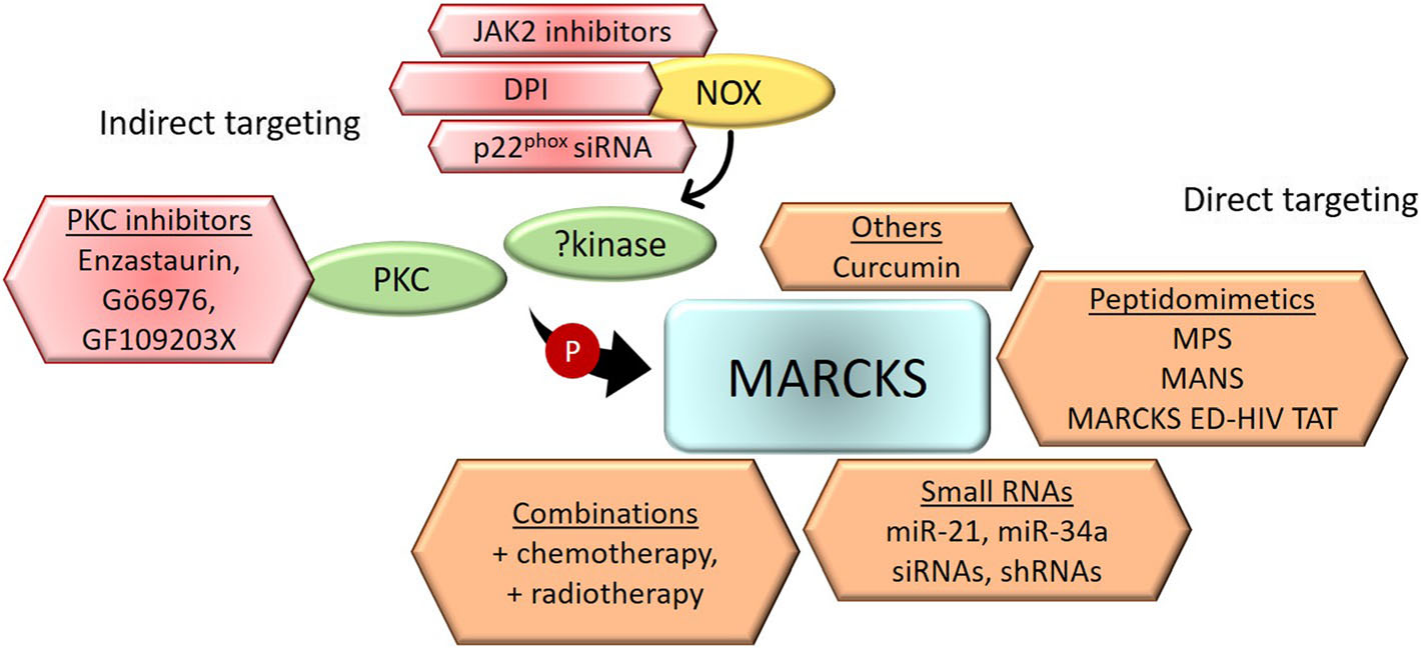
Strategies to target MARCKS signaling. Schematic depicting the current approaches available to target MARCKS signaling in cancer. Indirect targeting includes inhibitors for PKC and NADPH oxidase (NOX) which induce the phosphorylation of MARCKS. Direct targeting of MARCKS can be performed using small RNAs, peptidomimetics, or alternative strategies as a single agent or in combination with traditional chemotherapy and/or radiotherapy

Compelling preclinical in vitro and in vivo evidence suggests that MARCKS plays a critical role in the development and progression of several hematological malignancies. Moreover, acquired resistance to proteasomal inhibitor-based therapy has been commonly shown to be associated with an increased expression of MARCKS [15, 132]. Hence, patients with “MARCKS-driven blood cancers” have a higher risk of disease progression or recurrence and an overall worse prognosis [14, 132, 161]. Consequently, targeting MARCKS, whether directly or indirectly, is a viable therapeutic alternative with landmark clinical repercussions for the treatment of several solid and blood cancers. Figure 3 summarizes the strategies explored until now to target MARCKS signaling in hematological malignancies.

### Direct targeting of MARCKS

Recently Curcumin was found to inhibit the expression of *MARCKS* in CML cells [119]. Curcumin is a well-researched polyphenol derived from the rhizome *Curcuma longa* that has anti-cancer effects in several malignancies. Among other molecular mechanisms, Curcumin has been shown to inhibit the PI3K/Akt signaling pathway [162] that has been shown to be indirectly regulated by MARCKS control of free PIP_2_ bioavailability. Furthermore, Curcumin has also been shown to impact the expression of miR-21 which subsequently translates the effect of the polyphenol derivative on the proliferation, growth, survival, and apoptosis of cancer cells [163]. As a proof of principle, exosomes released from CML cells treated with Curcumin were shown to contain a high concentration of miR-21 which also served as an efficient inhibitor of the expression of *MARCKS* in target cells [119]. Interestingly, MARCKS has been shown as a direct target for sequence-specific suppression by miR-21 [164]. Indeed, the importance of small non-coding RNA-based inhibition of genetic targets such as MARCKS has been recognized by several studies for its vital therapeutic consequence in multiple cancers. Results from our lab demonstrated that low levels of miR-34a are associated with an increased expression of MARCKS, higher resistance to anti-myeloma drugs, disease progression, and an overall poor prognosis in MM [132]. Furthermore, upregulated expression of the tumor-suppressive miR-34a was found to directly inhibit MARCKS in a sequence-specific manner and decreased the viability of drug-resistant cells upon treatment with MM-associated chemotherapeutic agents. Similar results were observed following the genetic knockdown of MARCKS by a synthetic siRNA molecule or a lentiviral shRNA [14, 132].

While small RNA-based therapeutics hold great promise, their clinical usage has been limited by several factors including their stability, precision-based delivery to the target site as well as timed release, off-target effects, and immune responses [165, 166]. Hence, several groups investigated the potential of alternative therapeutic strategies such as making use of peptides to target critical domains of MARCKS. Three MARCKS targeting peptidomimetics have been developed so far. These include two PSD peptidomimetics, MPS [167], and MARCKS ED-HIV TAT [168] that prevent the phosphorylation of MARCKS, and a myristoylated *N-*terminal targeting MANS peptide [169]. Whereas MPS has proven to be highly effective in suppressing the progression of several cancers [106, 170], MARCKS ED-HIV TAT is particularly potent in sensitizing progressive lung cancer to radiation therapy [168, 171], and MANS has been shown to efficiently repress metastasis development in the lung [108] and breast [114] cancers. Recently our lab made use of these peptidomimetics for the very first time to target MARCKS in blood cancer. Specifically, we demonstrated a dose-dependent cytotoxic effect of the PSD peptidomimetic MPS in drug-resistant MM cells in vitro and in vivo, caused by the targeted inhibition of MARCKS phosphorylation [132]. In contrast, MPS was well tolerated by the normal hematopoietic cells, indicating the high specificity of the peptide for MM cells. Interestingly, MPS-induced suppression of phosphorylated-MARCKS caused the activation of several key members of the autophagy pathway including Microtubule-Associated Protein 1 Light Chain 3 Beta (MAP1LC3B), CDK1B/p27^kip1^, and PUMA, all of which have the potential to play a key role in the survival of MM cells. Consequently, our findings advocated the usage of a synergistic combination of MPS, anti-MM drugs such as bortezomib, and an autophagy inhibitor like chloroquine to overcome disease resistance and achieve better control over the progression of MM, as compared to MPS alone. Surprisingly, MANS treatment did not cause significant suppression of cell proliferation in control or drug-resistant MM cell lines in our study, suggesting that the biological roles of MARCKS in MM are possibly myristoylation-independent, and are primarily enunciated through the activity of the PSD. This indeed is an indicator of the evolving nature of our understanding of the structural and functional roles of MARCKS. In fact, recently a biopharmaceutical company developing anti-MARCKS technology demonstrated the highly promising clinical efficacy without any serious adverse events of a novel peptidomimetic BIO-11006 that blocks MARCKS phosphorylation, from a phase 2 study in non-small cell lung cancer [172]. Although, a probable therapeutic constraint may be observed in blood cancers which are driven by the unphosphorylated form of MARCKS. Nonetheless, these pre-clinical investigations have shown that MARCKS can serve as a druggable target in MM, although further validation in other hematological malignancies will add value to the clinical application of several such MARCKS-targeting inhibitors as potential therapeutic agents in blood cancers.

### Indirect targeting of MARCKS

Considering that majority of cancer-related studies have described an oncogenic role of MARCKS to be associated with its phosphorylated form, inhibiting the molecular factors that directly or indirectly induce the phosphorylation and activation of MARCKS is a possible therapeutic strategy in improving disease response. Reddy et al. demonstrated that p22^phox^, a critical subunit of NADPH oxidase 1–4, played an important role in the phosphorylation of MARCKS which subsequently was observed to cause an increased migration of patient-derived myeloid cells [120]. Treatment of the cells with a siRNA targeting p22^phox^ or a flavoprotein inhibitor diphenyleneiodonium to block the function of NOX resulted in the reduced phosphorylation of MARCKS as well as a significant decrease in the rates of cell migration. Similar results were observed in other studies that attempted to target PKC with the objective of inhibiting the phosphorylation of MARCKS. Gutiérrez et al. demonstrated that MARCKS is overexpressed in clonal B-lymphocytes isolated from Waldenström’s macroglobulinemia, a very rare form of B-cell NHL [127]. Interestingly, aberrant PKC signaling has been reported as one of the prime contributors to tumor progression and increased cell survival of B-cell lymphoma [173, 174]. Treatment with selective PKC-inhibitor such as Enzastaurin which also inhibits the PI3K signaling pathway has been shown to cause a time- and dose-dependent blockage of phosphorylation of MARCKS, GSK3β, and ribosomal S6 kinase [16]. The net result is a significant reduction in tumor cell growth, cell proliferation, and induction of apoptosis, thereby indicating the potential of PKC-inhibitors such as enzastaurin in the treatment of B-cell lymphomas. Likewise, Podar et al. demonstrated the effectiveness of the PKC-inhibitor enzastaurin in blocking the phorbol ester-induced activation of several PKC isoforms while concurrently disrupting the phosphorylation of downstream signaling molecules MARCKS and PKCμ in MM [131]. Consequently, enzastaurin was shown to inhibit key oncogenic signals governing cell proliferation, survival, angiogenesis, and migration in MM, proving its efficacy as a vital therapeutic agent for clinical evaluation. Although targeting PKC may be worthwhile as a means of indirectly inhibiting the function of MARCKS, clinical application of this technique has been difficult considering the existence of multiple isoforms of PKC. Moreover, inhibiting PKC is also associated with the risk of disrupting the function/s of other downstream targets that may not be associated with MARCKS. Indeed, Muguruma and colleagues reported that selective targeting of PKC isoforms using enzastaurin (inhibits PKCβ) or Gö6976 (inhibits PKCα and β) proved insignificant in rescuing Jagged1-mediated phosphorylation of MARCKS and consequently the enhanced survival of bortezomib-resistant MM cells [18]. Although, the addition of GF109203X, a pan-PKC inhibitor was found to efficiently decrease the viability of myeloma cells, thereby suggesting that PKCs associated with MARCKS activation and drug resistance may be context-dependent in view of the heterogeneity of the pathophysiology among the patients [18]. Additionally, cancers that are driven primarily by the unphosphorylated form of MARCKS [15] may not find a significant role of PKC inhibitors. Under such circumstances, direct-targeting of MARCKS will have a greater clinical impact and this will also reduce any off-target effects that may result from the inhibition of PKC.

## Conclusion

Although we are at a very early stage to draw a conclusion, the body of knowledge that we have until now demonstrates that MARCKS is strongly involved in the development and progression of several blood cancers. Further research into understanding the molecular network of MARCKS and its functional significance is essential owing to the existence of several strongly conflicting evidence lines that have indicated an oncogenic versus tumor-suppressive function of MARCKS in multiple hematological malignancies. In addition, the impact of phosphorylated MARCKS versus the unphosphorylated form of MARCKS or the membrane-bound MARCKS versus the cytosolic MARCKS on the overall process of carcinogenesis lacks clarity. Nevertheless, the current data is indicative of a vital role of MARCKS in regulating the risk factors associated with the global carcinogenesis process, mediating resistance to therapy, and determining the overall outcome of the disease. Interestingly, the role of MARCKS as a prognostic biomarker in blood cancers has not been explored significantly, despite its strong involvement in therapeutic resistance. While some of the current strategies to reverse the abnormal function of MARCKS are promising, significant research is required to advance them for clinical use. Moreover, there are a very limited number of studies that have developed or incorporated animal models for exploring the biology of MARCKS in vivo. Undeniably, the development of animal models would tremendously benefit the pre-clinical research studies investigating the efficacy of new therapeutic strategies targeting MARCKS. Indeed, with an improved structural and functional understanding of MARCKS, there exists a strong potential to develop novel therapies to better tackle MARCKS-driven pathophysiology in hematological malignancies.

## Data Availability

Data sharing is not applicable to this article as no datasets were generated or analysed during the current study.

## Abbreviations

MARCKS: Myristoylated Alanine-Rich C-Kinase Substrate
PKC: Protein Kinase C
NMT: N-MyristoylTransferase
MH2: MARCKS Homology 2
CI-MPR: Cation-Independent Mannose-6-Phosphate Receptor
PSD: Phosphorylation Site Domain
ED: Effector Domain
ROCK: Rho-associated kinase
MRP: MARCKS-Related Protein
MLP: MARCKS-Like Protein
MARCKSL1: MARCKS-Like protein 1
CaM: Calmodulin
CDK1: Cyclin-Dependent Kinase 1
TPKII: Tau Protein Kinase II
F-actin: Filamentous actin
PIP_2_: Phosphatidylnositol-4,5-bisphosphate
PLC: Phospholipase C
IP_3_: Inositol-1,4,5-trisphosphate
DAG: Diacylglycerol
N-WASP: Neural-Wiskott-Aldrich Syndrome Protein
PA: Phosphatidic acid
PI3K: Phosphoinositide-3-kinase
PTK: Protein tyrosine kinase
TOB2: Transducer of ERBB2, 2
DCC: Deleted in Colorectal Cancer
B-CLL: B-chronic lymphocytic leukemia
LSP1: Leukocyte-specific protein 1
EBV: Epstein-Barr virus
BL: Burkitt Lymphoma
EBNA2: EBV nuclear antigen 2
LCL: Lymphoblastoid cell lines
LMP: Latent membrane protein
MCL: Mantle cell lymphoma
NHL: Non-Hodgkin’s lymphoma
DLBCL: Diffuse large B-cell lymphoma
AML: Acute myeloid leukemia
MM: Multiple Myeloma
MAP 1 LC3B: Microtubule-Associated Protein 1 Light Chain 3 Beta
CDK1B: Cyclin-dependent kinase inhibitor 1B
PUMA: p53 upregulated modulator of apoptosis
